# Improvement of Dye-Sensitized Solar Cell Performance via Addition of Azopyridine Derivative in Polymer Gel Electrolytes

**DOI:** 10.3390/ma17246107

**Published:** 2024-12-13

**Authors:** Muhammad Faisal Amin, Paweł Gnida, Jolanta Konieczkowska, Magdalena Szubka, Ewa Schab-Balcerzak

**Affiliations:** 1 Centre of Polymer and Carbon Materials, Polish Academy of Sciences, 34 M. Curie-Sklodowska Str., 41-819 Zabrze, Poland; mfaisal-amin@cmpw-pan.pl (M.F.A.); jkonieczkowska@cmpw-pan.pl (J.K.); 2 Joint Doctoral School, Silesian University of Technology, Akademicka 2a, 44-100 Gliwice, Poland; 3 A. Chełkowski Institute of Physics, University of Silesia, Uniwersytecka 4, 40-007 Katowice, Poland; magdalena.szubka@us.edu.pl; 4 Institute of Chemistry, University of Silesia, 9 Szkolna Str., 40-007 Katowice, Poland

**Keywords:** polymer gel electrolyte, dye-sensitized solar cells, AZO dyes

## Abstract

In this study, a polymer gel electrolyte based on polyacrylonitrile was synthesized with varying polymer-to-liquid-electrolyte ratios. DSSCs incorporating a 1:3 ratio showed optimum PV parameters. Choosing this proportion, the effect of incorporating the photoresponsive AZO dye into this polymer electrolyte was studied. When irradiated with a UV light of 365 nm, the AZO dye underwent photoisomerization, which allowed the gel electrolyte to absorb heat from the UV irradiation and increase its ionic conductivity. It was found that by the addition of azopyridine into the polymer electrolyte, there was an improvement in the photovoltaic parameters of cells. By increasing the dye content from 1% to 10% by weight in the electrolyte, an 11% growth in short current density was observed, resulting in about a 10% rise in cell efficiency.

## 1. Introduction

An electrolyte is the most indispensable component of dye-sensitized solar cells (DSSCs) as it contains a redox mediator (e.g., I^−^/I_3_^−^) and helps in the regeneration of the oxidized dye during the operation of the device. Until now, various types of electrolytes, including liquid, solid-state, and polymer gel electrolytes, have been prepared for DSSCs. DSSCs incorporating liquid electrolytes show the best PV parameters as compared to the other two types. However, certain unavoidable issues have been associated with liquid electrolytes, making them lethal not only for the device’s efficiency but also mainly for the device’s stability [1]. The use of low-boiling solvents in liquid electrolytes and, consequently, their low density cause not only leakage but also evaporation of the electrolyte during longer device operation times, thus decreasing device stability. Moreover, liquid electrolytes are corrosive towards cell components [2,3]. To avoid the shortcomings associated with the use of liquid electrolytes, first, solid-state electrolytes were introduced long ago [4]. However, it was found that solid electrolytes also have certain disadvantages such as lower ionic conductivity and weaker interfacial contact between the two electrodes [5]. Polymer gel electrolytes, however, are potential substitutes for liquid and solid-state electrolytes in dye-sensitized solar cells due to their high ionic conductivities, excellent interfacial filling ability as compared to solid-state electrolytes, and non-propensity to electrolyte leakage. The idea of a quasi-solid-state or polymer gel electrolyte (PGE) was put forth in 1995 by Cao F. et al. [6].

Since then, PGEs incorporating various polymers such as polyacrylonitrile (PAN), poly(methyl acrylate) (PMA), poly(methyl methacrylate) (PMMA), poly(ethylene oxide)-poly(vinylidene fluoride) (PEO-PVDF), and poly(vinylidene fluoride-co-hexafluoropropylene) (PVDF-HFP) have been well documented [7,8,9,10,11]. Dong, R-X., for instance, compared polymer gel electrolytes with liquid electrolytes [12]. The electrical conductivity of the polymer gel electrolyte was 12.77 ± 0.11 mScm^−1^, which was even higher than that of the liquid electrolyte, i.e., 12.70 ± 0.07. The power conversion efficiency (PCE) in the former case was 9.48% while it was 8.84% in the case of the liquid electrolyte. Jayaweera, E.N. et al. reported that the interfacial pore filling ability of PGEs can be further enhanced by incorporating liquid electrolytes as redox pairs [13]. They obtained a high PCE of 8.4% by incorporating a liquid electrolyte in PGEs as compared to that of PGEs without a liquid electrolyte, i.e., only 4.1%. The former value was close to 9.8%, which was obtained using a liquid electrolyte alone.

**Figure 1:**
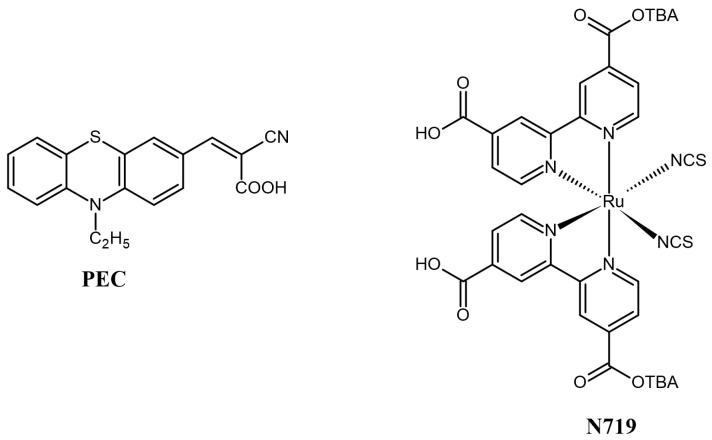
Chemical structure of the dyes used in this study.

Among the numerous polymers available, polyacrylonitrile has been widely studied to prepare polymer gel electrolytes [14,15]. It has a conductivity value of above 1 mScm^−1^, and the lone pair present on the nitrogen atom of the nitrile group can form an interaction with the cation of the salt in the polymer electrolyte [16]. Since the first use of PGEs for dye-sensitized solar cells in 1995, various researchers have tried to improve the efficiency and stability of DSSCs using PAN-based gel electrolytes [17,18,19]. However, due to the high viscosity of polymer gel electrolytes, redox pair ions feel resistance during their movement, and therefore, the ionic conductivity is low, which directly affects the photovoltaic parameters. A plausible solution to this problem lies in the fact that the viscosity of the gel electrolyte can be momentarily decreased by the absorption of heat energy mainly in the form of infrared light, which is in accordance with the Vogel–Tamman–Fulcher (VTF) equation, and hence, the ionic conductivity can be increased [20,21]. Polyacrylonitrile is a colorless polymer which reflects the majority of infrared radiations, and therefore, it is difficult to obtain a high-temperature polymer electrolyte using pristine PAN [22]. However, the absorption of light by the polymer can be increased by the addition of photoresponsive dyes such as azobenzene to the polymer gel electrolyte, which results in a stimulus-responsive polymer gel [23,24,25]. Huang, Y. et al. synthesized a nanostructured photo-heatable polyacrylonitrile-based GPE and tested it in dye-sensitized solar cells [26]. In situ imaging using an IR imaging system revealed that when the electrolyte C11-AZO-C11/PAN (I^−^/I_3_^−^) was exposed to light at 365 and 450 nm, the temperature of the electrolyte increased from 25 °C to 32 °C, while in the case of the electrolyte without AZO dye, the temperature only fluctuated around 25 °C. Correspondingly, the conductivity of the dyed electrolyte increased from 1.0 mScm^−1^ at 26.2 °C to 1.4 mScm^−1^ at 34 °C. It is due to this rise in the conductivity of the azobenzene-containing electrolyte that the device incorporating this electrolyte showed a PCE of 6.28% (comparable to that of the device using a liquid electrolyte, namely, 6.30%), which was much higher compared to that of the device using a PAN electrolyte without added dye, i.e., 4.19%. Inspired by these results, in this study, a series of polyacrylonitrile-based gel electrolytes were first synthesized by varying the concentration of liquid electrolyte in them. To the best of our knowledge, phenothiazine and N719 dye have never been tested for such optimizations. The prepared electrolytes were tested in DSSC devices sensitized with N719- and phenothiazine-based donor/acceptor dye (2-Cyano-3-(10-ethyl-10H-phenothiazin-3-yl)acrylic acid (PEC)) (cf. Figure 1). In these cases, a maximum PCE of 1.77% and 1.73% was obtained for N179 and PEC dye, respectively, when the polymer-to-liquid-electrolyte ratio was 1:3. Following this optimization, an azopyridine dye (AZO) was added in varying amounts into the electrolyte containing an optimized ratio of polymer to liquid component, i.e., 1:3. The weight percentage of the azopyridine derivative was varied between 1% and 10% to study the effect of the content of the AZO dye in the PGE on the photovoltaic parameters of the dye-sensitized solar cells. After the addition of 10% AZO dye, the power conversion efficiency of the PGE was improved by 16% compared to that without the AZO dye.

## 2. Experimental

### 2.1. Materials and Measurements

4-Aminopyridine, phenol, and sodium nitrate, were purchased from Merc. Hydrochloric acid and sodium hydroxide were purchased from POCH. N,N-dimethylformamide (DMF), acetonitrile (ACN), tert-butanol, liquid electrolyte EL-HSE, benzene, chloroform ethanol, N-methyl pyrrolidone (NMP), dimethyl sulfoxide (DMSO), polyacrylonitrile, and FTO glass substrates were purchased from Sigma Aldrich (St. Louis, MO, USA). The FTO substrates had a thickness of 2.3 mm, sheet resistance of ~7 Ω/sq, roughness of 32 nm, and transmission of 80–82% (visible). Di-tetrabutylammonium cis-bis(isothiocyanato)bis(2,2′-bipyridyl-4,4′-dicarboxylato)ruthenium(II), **N719** dye was purchased from Sigma Aldrich (St. Louis, MO, USA). 18NR-T transparent titania paste was purchased from 18NR-T, Greatcell Solar Materials (Queanbeyan, Australia). The synthesis of 2-cyano-3-(10-ethyl-phenothiazine-3-yl)-acrylic acid was performed as described in the paper [27].

The absorption studies of the AZO dye were performed using a Jasco V-750 spectrophotometer (Jasco Inc., Tokyo, Japan). The PV parameters of the dye-sensitized solar cells were determined using a PV Solutions solar simulator and a Keithley 2400 SourceMeter (Tektronix, Inc., Beaverton, OR, USA) under AM 1.5 G illumination (100 mWcm^−2^).

### 2.2. Synthesis of Azopyridine Dye (AZO)

**Figure 2:**
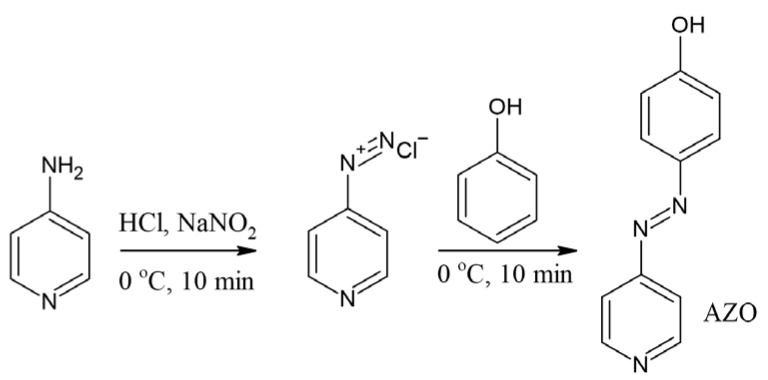
Synthesis scheme of AZO dye.

The 4-(4-hydroxyphenylazo)pyridine (denoted as AZO—see Figure 2) was synthesized and characterized in our previous work [28].

4-Aminopyridine (30 mmol) was dissolved in a mixture of deionized water (40 mL) and concentrated HCl (18 mL). The solution was stirred in an ice bath (0–5 °C), and a solution of sodium nitrate (30 mmol) in 5 mL of deionized water was added dropwise. Next, a solution of phenol (30 mmol) in 50 mL of acetone was added dropwise to the solution of 4-aminopyridine. The mixture was stirred for 30 min. Finally, the mixture was further neutralized to pH = 6–7 by adding a 10% solution of sodium hydroxide. The product was filtrated and washed with deionized water. The yield of the reaction was 89%.

### 2.3. Preparation of Polymer Gel Electrolyte

**Figure 3:**
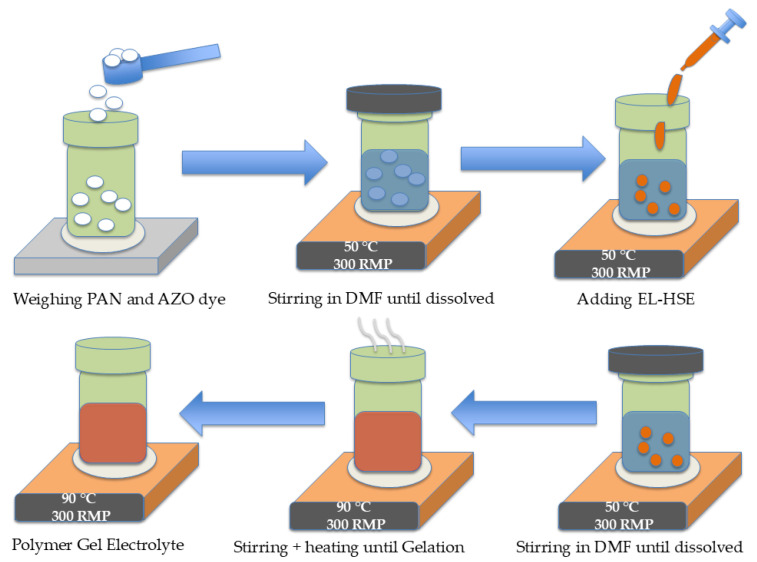
Schematic representation of PGE synthesis without AZO dye.

First, 1.0 g of polyacrylonitrile without AZO was dissolved in 12.5 mL of DMF at 50 °C, and the resulting mixture was stirred for 6 h. After a clear solution was obtained, the calculated amount of EL-HSE electrolyte was added. A series of five different polymer gel electrolytes with polymer-to-EL-HSE ratios of 1:1, 1:3, 1:5, and 1:7 were prepared by repeating this procedure. The scheme of synthesis is shown in Figure 3. To synthesize the photoresponsive AZO dye-containing PGE, the optimum mass ratio of composite to liquid component was selected as 1:3. AZO dye was added along with polyacrylonitrile in DMF. After stirring the solution for 6 h, 2.5 mL of ethanol was added to the mixture, resulting in the precipitation of product. The precipitates were then filtered, dried, and ground in a ball mill. After grinding, the resulting product was placed in a vacuum dryer and the whole product was dried again for 24 h at 40 °C. The final step was to place the resulting nanostructures in EL-HSE liquid electrolyte for 24 h to obtain a gel electrolyte.

### 2.4. Fabrication of DSSCs

**Figure 4:**
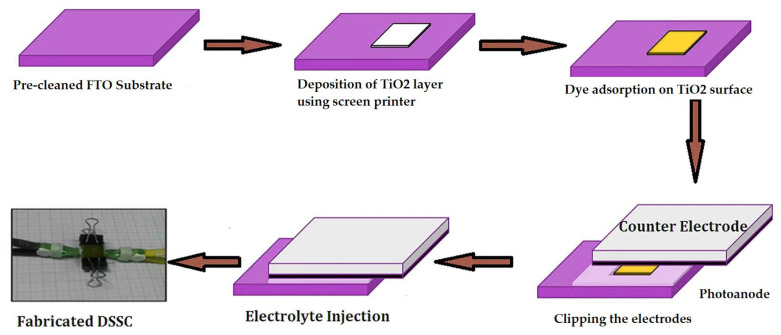
Schematic representation of dye-sensitized solar cell fabrication process.

**Figure 5:**
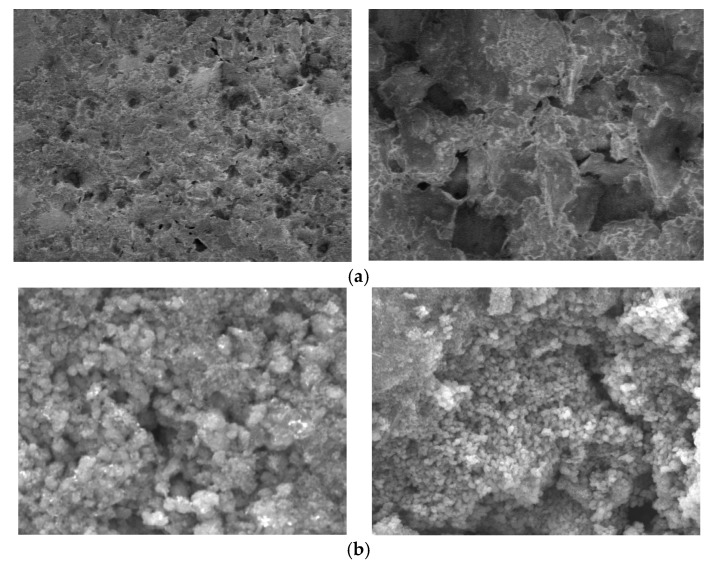
SEM pictures of the (**a**) azopyridine-derived polymer matrix morphology during electrolyte preparation and (**b**) photoanode.

Prior to use, the FTO substrates were cleaned by sonicating using a commercial detergent, distilled water, and isopropanol for 15 min each at 40 °C. After drying, the substrates were screen-printed with titanium dioxide layers and were annealed at 500 °C for 30 min. For the preparation of TiO_2_ layers, commercially available TiO_2_ paste (18NR-T) was used, which contained anatase TiO_2_ nanoparticles with an average size of 20 nm. In previous studies conducted by our group, it was found that using TiO_2_ paste (18NR-T) and baking in a furnace at 500 °C results in a crystalline structure of anatase, which is very important due to the higher number of hydroxyl groups to which dye molecules can anchor compared to rutile [29]. The scheme of fabrication of the DSSCs is shown in Figure 4. The thickness of the TiO_2_ layers was approximately 8.5–9 µm. The surface roughness, expressed as RMS, for the FTO/TiO_2_ substrate (before dye adsorption) was about 70 nm, which decreased to about 40–50 nm after dye adsorption for the FTO/TiO_2_@N719 photoanode. This decrease in RMS value after the dye molecules were adsorbed indicates that they filled the pores in the TiO_2_ structure well and there was a smoothing of the photoanode surface [29]. Figure 5 shows the change in morphology of the azopyridine-derived polymer matrix during electrolyte preparation. The FTOs covered by TiO_2_ were immersed in **N719** or **PEC** dye solution (3 × 10^−4^ moldm^−3^) prepared using an acetonitrile/tert-butanol (1:1) solvent mixture. After 24 h, the substrates, now called photoanodes, were removed from the solution and washed with ethanol. The gel electrolyte was applied before clamping the platinum counter electrode with the photoanode to assemble the DSSCs.

## 3. Results and Discussion

**Figure 6:**
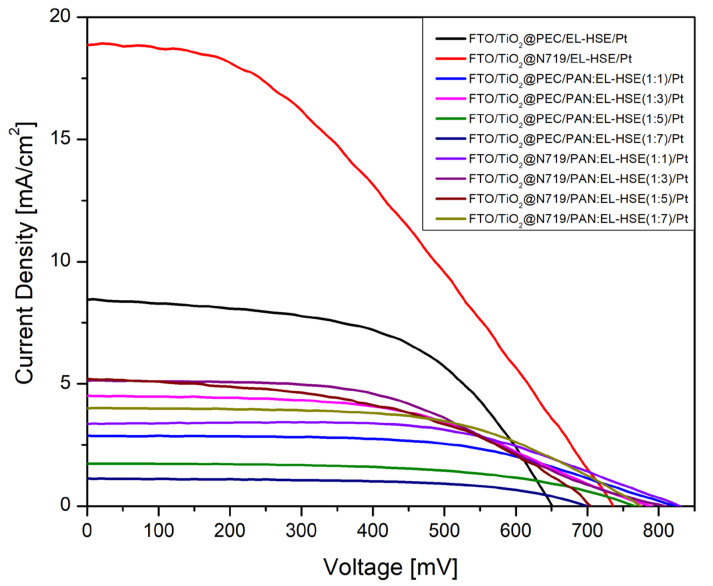
J–V curves of dye-sensitized solar cells incorporating PGEs of varying redox concentrations.

As mentioned earlier, the DSSCs were sensitized with the metal-free all-organic dye **PEC** and the organometallic dye **N719**. The dye N719 was chosen because it is a commercial compound and is often used as a reference dye in reference cells, including by our research group. It is also often used by us for preliminary research on introducing new modifications. In the case of PEC dye, previous studies [30] showed that among the obtained dyes in our research group, the cell containing PEC achieved the highest PV parameters. Therefore, PEC was chosen for testing further structural modifications of DSSCs as a metal-free dye. The purpose was to conduct optimization studies for polymer gel electrolytes which will be suited to both types of dyes. For each type of measurement, three DSSC devices were fabricated, and the resulting photovoltaic parameters are summarized in Table 1, while current density–voltage (J–V) curves are presented in Figure 6. It can be noted from Table 1 that the highest PV parameters were obtained when a liquid electrolyte was used. The electrolyte has a direct impact on the open circuit voltage (V_OC_) of DSSCs because the V_OC_ is mainly determined by the difference between the Fermi level of the metal oxide on which dye is adsorbed and the redox potential of iodide-triiodide ions in the electrolyte. This redox potential in turn depends on the composition of the electrolyte. It is interesting to note that the open circuit voltage of the devices incorporating polymer gel electrolytes was continuously higher than that of those incorporating liquid electrolytes, irrespective of which type of dye was used.

However, the definite decrease in J_sc_ was responsible for the lower efficiencies of the solar cells containing gel electrolytes. This is likely due to a reduction in ion transport efficiency within the electrolyte, leading to an increase in layer resistance. For both solar cells containing **N719** dye and **PEC**, the PAN/EL-HSE ratio of 1:3 was the most favorable in terms of the efficiencies obtained, reaching 1.77 and 1.73%, respectively. The reason for obtaining maximum PV parameters at the 1:3 ratio of polymer to EL-HSE can be attributed to the fact that the concentration of the redox couple in the electrolyte has a direct impact on the conductivity of the electrolyte. Shen, S.-Y., et al. also varied the concentration of electrolytes in polymer gel electrolytes [31]. They found that the conductivity of the electrolyte increased steeply up to a certain limit and then started to decrease. The reason was attributed to the high viscosity of the polymer gel electrolyte by increasing the concentration of iodide triiodide. Abrol, S.A., et al. also varied the proportion of polymer to liquid electrolyte and found that the conductivity only increases up to a certain ratio and then starts to decrease [32]. The electrolyte with this optimal ratio showed the best PV parameters. It is noteworthy that the FF values in both cases in our study were comparable and often even higher when using the gel electrolyte, especially when using **N719** dye, indicating their good quality. Figure 6 shows the J–V curves of DSSCs fabricated using liquid and polymer gel electrolytes.

**Figure 7:**
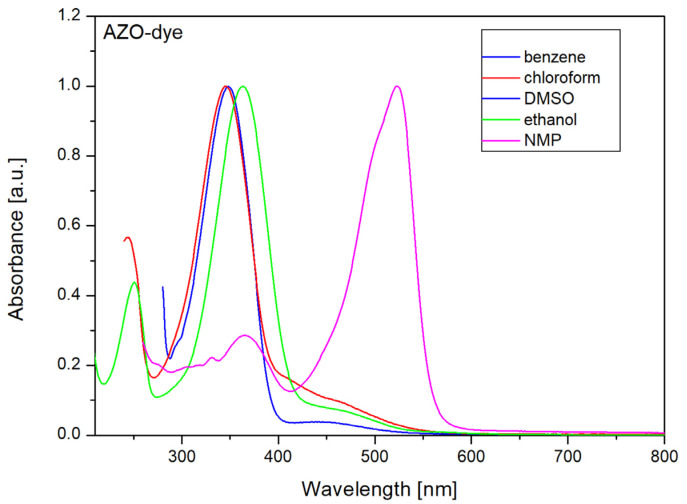
UV-vis spectra of AZO dye in different solvents (c = 10^−5^ mol/L).

Similar studies on polyacrylonitrile-based polymer gel electrolytes for DSSCs have been published, demonstrating how electrolyte composition affects the overall performance of dye-sensitized solar cells. Akhtar, M.S., et al. incorporated carbon nanotubes (CNTs) during the electropolymerization of polyacrylonitrile and polymethylmethacrylate [33]. They introduced 0.1 M lithium iodide (LiI), 0.015 M iodine (I_2_), and 0.2 M t-butyl pyridine (TBP). The detailed PV parameters obtained are compiled in Table 2, showing that the PGE based on polyacrylonitrile had a better performance due to the higher conductivity of this electrolyte. When the composition of the electrolyte was changed, the PV parameters changed. Table 2 further shows the PV parameters when lithium iodide was replaced by magnesium iodide (MgI_2_) and t-butyl pyridine was replaced by propylene carbonate (PC) and ethylene carbonate (EC) [34,35]. This implies that when the composition of the electrolyte was changed, the photovoltaic performance was directly changed. In addition to conductivity, the composition change can also alter the pH of an electrolyte, which also affects the performance of DSSCs. The components of DSSCs are highly prone to pH change. For instance, a high pH in an electrolyte can not only corrode the titanium dioxide surface but also degrade the dye [36]. Besides modifying the core composition, one can also add secondary components such as photoresponsive dyes, which can also increase the performance of DSSCs incorporating such PGEs. Therefore, a further step in this research was also conducted to modify the gel electrolyte by adding an azobenzene derivative. The polymer gel electrolyte was further modified by reducing the size of the particles to nanoscale by the addition of AZO dyes. Based on previous results, **N719** dye was chosen for further testing due to the fact that the solar cell in which it was used showed a higher efficiency than that using **PEC** dye. Figure 7 shows the UV-vis absorption spectra of the AZO dye in benzene, chloroform, DMSO, ethanol, and NMP solutions (c = 10^−5^ mol/L).

The UV-vis spectra show two absorption bands. The high-intensity band located at 365 nm is attributed to the π–π* transition, while the low-energy absorption signal at around 450 nm is attributed to the n–π* transition for the trans-isomer. In the NMP solvent, a higher intensity of the absorption band was observed at 450 nm. It could be the result of changes in electronic distribution in the molecule as a consequence of the intermolecular H-bonds (NMP-dye). Trans-cis-trans isomerization of AZO dye can be generated by UV-light irradiation. The kinetics of cis-trans isomerization for AZO dye were studied in previous works [37,38]. The value of the k_c-t_ isomerization rate is dependent on the used solvent, i.e., 1.3 × 10^−2^ 1/s in THF and 1.7 × 10^−1^ 1/s in chloroform. Fast recovery of the trans-isomer was observed in ethanol, where the relaxation time was 14 × 10^−3^ s [39].

The photoisomerization of AZO dye is caused by the absorption of heat energy coming from the incident light; thus, when incorporated into the polymer matrix, the heat energy absorbed by this dye expands the volume of the gel electrolyte, causing its viscosity to decrease and resulting in the increase in the conductivity of the redox ion pairs in the polymer matrix. The rate of interconversion of the two isomers of the dye greatly depends on how well it interacts with the polymer matrix [40]. In this study, we prepared two types of PAN-based polymer gel electrolytes, where the iodide/triiodide couple was used as a redox pair and the weight percentage of AZO dye was varied between 1 and 10 percent. One such gel electrolyte was prepared without added dye for comparison purposes. These gel electrolytes were used to fabricate three quasi-solid-state dye-sensitized solar cells based on commercial **N719** dye. The photovoltaic parameters of the DSSCs obtained are compiled in Table 2.

The results show that the AZO dye interacted well with PAN (I^−^/I_3_^−^), which resulted in a higher interconversion rate of the two isomers of the dye; thus, a greater amount of heat was absorbed and transferred by the dye to the polymer chains. This increase in heat in the polymer gel eventually increased its ionic conductivity, which was obvious in the higher PV parameters in the case of the PAN/AZO(10%)/I^−^/I_3_^−^ electrolyte [40].

It can be noted that there was a significant increase in the value of open circuit voltage by the addition of AZO dye to the gel electrolyte as compared to the electrolyte without AZO. This enhancement is attributed to the increased ionic conductivity of the gel electrolyte, as the dye absorbs heat energy, facilitating the quick regeneration of the oxidized form of the dye through the redox couple. The increase in the conductivity of gels by the addition of azobenzene is also evident from many studies [25,40]. However, the short-circuit current density was not affected much by the addition of 1 weight percent of AZO dye. Nevertheless, when the weight percentage of the AZO dye was increased, the value of the J_SC_ increased from 3.84 to 4.28 mAcm^−2^, thus producing an increase in the PCE of this device by about 10% as compared to the PCE obtained using the PGE incorporating 1 weight percent of the dye (cf. Table 1).

**Figure 8:**
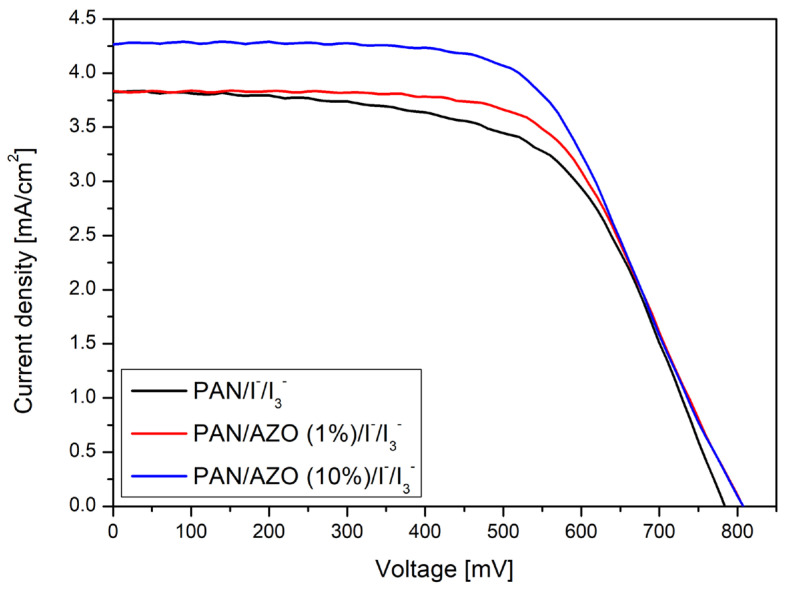
Current density–voltage characteristics of the prepared DSSCs.

Figure 8 shows the J–V curves of DSSC devices prepared using polymer gel electrolytes. The curve representing PAN/I^−^/I_3_^−^ is the lowest and has the shortest covering along the *x*-axis, which means that the gel electrolyte without any added dye had the lowest values for current density and open circuit voltage, i.e., 3.83 mAcm^−2^ and 784 mV, respectively. In contrast, the curves for PAN/AZO(1%) and (10%)/I^−^/I_3_^−^ are both higher and wider, which implies that the addition of AZO dye captured heat energy from the incident line and caused the gel electrolyte to decrease in viscosity, which helped the redox mediator ions to move faster in the electrolyte medium. This in turn helped to regenerate the oxidized form of the **N719** dye, and thus, a greater number of photoexcited electrons could be produced and transferred to the conduction band of the titanium dioxide, thereby increasing the J_SC_ and V_OC_ of the device to 4.28 mAcm^−2^ and 808 mV, respectively. However, the fill factor (FF) value was not significantly affected by the addition of the AZO dye. Thus, the overall power conversion efficiency of the DSSC incorporating the PAN/AZO(10%)/I^−^/I_3_^−^ electrolyte was measured to be about 2.06%, the highest among all of the three devices. The use of azopyridine or azobenzene derivatives influences the conductivity change and photovoltaic parameters in two ways. On the one hand, the cis-to-trans and cis-to-trans isomer transition under the influence of light absorption results in a local temperature increase in the electrolyte volume. This is due to rapid transitions between isomers, which can be likened to oscillations, accompanied by the release of energy as heat, which promotes charge transport. On the other hand, the change in cis-trans isomerism, during which the size of the molecule changes, can induce photoreactive ion conduction in the matrix [26].

Generally, all of the research in the field of PGEs is currently focused mainly on device performance, and the long-term stability of DSSCs is being overlooked. To commercialize and industrialize this technology, several issues must be addressed, including the sealing process when using polymer gel electrolytes, improving electrolyte conductivity, and ensuring reproducibility. Moreover, the efficiency of DSSCs is still too low as compared to commercial silicon-based solar cells; therefore, it is necessary to obtain a PCE over 17% to compete with commercial silicon solar cells [41]. Until now, only a single study has been reported where, instead of a single DSSC device, a whole solar module was prepared from DSSCs incorporating PAN-based polymer gel electrolytes [42]. An average short-circuit current density of 6.4 mAcm^−2^, an open circuit voltage of 540 mV, and an overall PCE of 3.7% were obtained from this module under 8 Wm^−2^. This solar module was capable of running small electronic devices such as calculators for several weeks. This shows that polyacrylonitrile-based polymer gel electrolytes can have a bright future once the aforementioned problems are addressed.

## 4. Conclusions

The redox pair concentration in a polymer gel electrolyte was optimized, and it was later incorporated with 4-(4-hydroxyphenylazo)pyridine, an AZO dye synthesized with good yield. By varying the ratio of polymer to liquid components in the electrolyte, the highest efficiencies of 1.77% (**N719**) and 1.73% (**PEC**) were achieved for the electrolyte containing a 1:3 ratio of polymer to EL-HSE (*w/w*). At this ratio, the AZO dye was incorporated into the polyacrylonitrile-based gel electrolyte at 1% to 10% by weight to study the effect of its content on PV device performance. DSSCs with the PGE based on PAN and containing 1 weight percent AZO dye showed a PCE similar to that of the device without AZO. However, increasing the AZO content in PAN to 10 weight percent resulted in PCE growth to 2.06%, which is 10% higher as compared to the reference cell. This research paves the way to further optimize the performance of dye-sensitized solar cells based on PAN-based polymer gel electrolytes. Future work will involve adding more types of AZO dyes to assess how the dye structure influences DSSC performance. Additionally, structural modifications to the DSSCs are expected to further enhance PV parameters. The research will also mainly focus on the precise characterization of gel electrolytes containing azobenzene or azopyridine derivatives. Important elements to consider will be the determination of thermal stability, stability under long-term exposure to sunlight, and conductivity with changes in the amount of added AZO dye.

## Figures and Tables

**Table 1:** Comparison of PV parameters of DSSCs incorporating liquid and polymer gel electrolytes with varying polymer-to-EL-HSE ratios.

Dye	Electrolyte	V_OC_ [mV]	J_SC_ [mAcm^−2^]	FF [-]	PCE [%]
**PEC**	Liquid EL-HSE	656 ± 10	8.27 ± 1.01	0.54 ± 0.04	2.92 ± 0.16
**PEC**	PAN:EL-HSE 1:1	802 ± 20	2.97 ± 0.27	0.54 ± 0.02	1.28 ± 0.10
**PEC**	PAN:EL-HSE 1:3	803 ± 13	4.53 ± 0.11	0.48 ± 0.03	1.73 ± 0.06
**PEC**	PAN:EL-HSE 1:5	770 ± 6	1.76 ± 0.47	0.55 ± 0.01	0.75 ± 0.23
**PEC**	PAN:EL-HSE 1:7	697 ± 18	1.15 ± 0.16	0.57 ± 0.03	0.46 ± 0.10
**N719**	Liquid EL-HSE	739 ± 6	18.65 ± 0.65	0.39 ± 0.02	5.26 ± 0.04
**N719**	PAN:EL-HSE 1:1	845 ± 13	3.56 ± 0.38	0.56 ± 0.03	1.69 ± 0.12
**N719**	PAN:EL-HSE 1:3	784 ± 8	3.83 ± 0.21	0.60 ± 0.01	1.77 ± 0.10
**N719**	PAN:EL-HSE 1:5	751 ± 39	4.15 ± 0.91	0.54 ± 0.06	1.65 ± 0.08
**N719**	PAN:EL-HSE 1:7	760 ± 15	3.97 ± 0.32	0.54 ± 0.03	1.63 ± 0.10

pH of electrolyte = 6; EL-HSE, commercial liquid electrolyte.

**Table 2:** Photovoltaic parameters of DSSCs with N719 obtained using AZO-based polymer gel electrolyte.

Electrolyte	V_OC_ [mV]	J_SC_ [mAcm^−2^]	FF [-]	PCE [%]	Ref.
PAN-CNT-LiI-TBP-ACN-I_2_	570	10.90	0.64	3.90	[33]
PMMA-CNT-LiI-TBP-ACN-I_2_	567	8.9	0.62	2.9	
PAN-EC-PC-MgI_2_-I_2_	704	4.10	0.68	2.00	[34]
PAN-EC-PC-MgI_2_-I_2_	684	6.82	0.55	2.56	[35]
PAN/I^−^/I_3_^−^	790	6.85	0.67	4.19	[26]
C11-AZO-C11/PAN (I^−^/I_3_^−^)	780	11.96	0.75	6.28	
PAN/I^−^/I_3_^−^	784 ± 8	3.83 ± 0.21	0.60 ± 0.01	1.77 ± 0.10	This work
PAN/AZO(1%)/I^−^/I_3_^−^	807 ± 10	3.84 ± 0.15	0.62 ± 0.01	1.88 ± 0.08	
PAN/AZO(10%)/I^−^/I_3_^−^	808 ± 6	4.28 ± 0.11	0.61 ± 0.01	2.06 ± 0.05	

## Data Availability

Data are contained within the article.
